# Supplementary material for: The Small RNA Universe of Capitella teleta
Source: Front Mol Biosci. 2022 Feb 25;9:802814. doi: 10.3389/fmolb.2022.802814 (PMC8915122; doi:10.3389/fmolb.2022.802814)
Supplement: Supplementary file 1 [file DataSheet1.ZIP › Supplement/known/CAPTEscaffold_591_25238.pdf]

Provisional ID : CAPTEscaffold_591_25238
Score total : 38474.9
Score for star read(s) : 3.9
Score for read counts : 38468
Score for mfe : 2.1
Score for randfold : 1.6
Score for cons. seed : -0.6
Total read count : 75465
Mature read count : 75434
Loop read count : 27
Star read count : 4

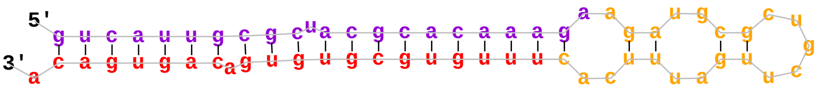

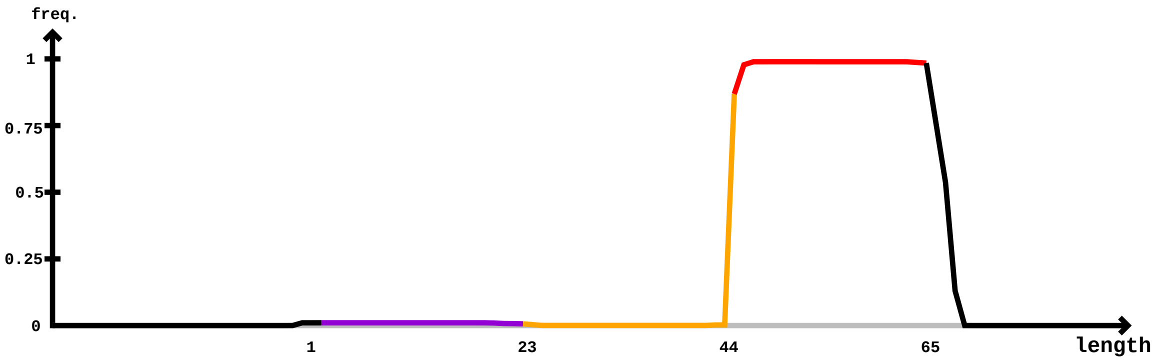


```
                                       Star                                       Mature
5'- cauuauaugagacggcgugugcguuggucauugcgcuacgcacaaagaagaugcgcugcuugauuucacuuugugcgugugacagugacaaugcacgcacgucacugcuu  -3'   obs
    cauuauaugagacggcgugugcguuggucauugcgcuacgcacaaagaagaugcgcugcuugauuucacuuugugcgugugacagugacaaugcacgcacgucacugcuu         exp
    cauuauaugagacggcgugugcguuggucauugcgcuacgcacaaagaagaugcgcugcuugauuucacuuugugcgugugacagugacaaugcacgcacgucacugcuu         known
    ..........((((..(((((((((.((((((((.(((((((((((..((..((.....))..))..))))))))))))).)))))))))))))))).)))).......   reads   mm   sample

    ......................guuggucauugcgcuacgcaca..................................................................   1       0    seq
    .......................uuggucauugcgcuacgcaca..................................................................   1       0    seq
    .......................Cuggucauugcgcuacgcacaaa................................................................   1       1    seq
    .......................uuggucauugcgcuacgcacaaag...............................................................   1       0    seq
    .......................uuggucauugcgcuacgcacaaaga..............................................................   2       0    seq
    ........................uggucauugcgcuacgcac...................................................................   5       0    seq
    ........................Nggucauugcgcuacgcac...................................................................   1       1    seq
    ........................uggucauugcgcuacgcaca..................................................................   54      0    seq
    ........................uggucauugcgcuacgcacG..................................................................   1       1    seq
    ........................uggucauugcgcuacgcacaa.................................................................   138     0    seq
    ........................uAgucauugcgcuacgcacaa.................................................................   1       1    seq
    ........................Aggucauugcgcuacgcacaa.................................................................   1       1    seq
    ........................uggucauugcgcuacgcacaaa................................................................   31      0    seq
    ........................uggucauugcgcuacgcacaaag...............................................................   69      0    seq
    ........................uggucauugcgcuacgcacaaagU..............................................................   1       1    seq
    ........................uggucauugcgcuacgcacaaagG..............................................................   1       1    seq
    ........................Aggucauugcgcuacgcacaaaga..............................................................   2       1    seq
    ........................uggucauugcgcuacgcGcaaaga..............................................................   1       1    seq
    ........................uggCcauugcgcuacgcacaaaga..............................................................   1       1    seq
    ........................uggucauugcgcuacgcacaaGga..............................................................   2       1    seq
    ........................uggucauugcgcuacgcacaaaga..............................................................   468     0    seq
    ........................Nggucauugcgcuacgcacaaaga..............................................................   1       1    seq
    ........................uggucauugcgcuacgcacaaagaU.............................................................   4       1    seq
    ..........................gucauugcgcuacgcacaaag...............................................................   1       0    seq
    ..........................gucauugcgcuacgcacaaaga..............................................................   3       0    seq
    ..............................................gaagaugcgcugcuugauuucacuu.......................................   2       0    seq
    ...............................................aagaugcgcugcuugauuucacu........................................   5       0    seq
    ................................................agaugcgcugcuugauuucac.........................................   20      0    seq
    ................................................agaugcgcugcuugauuucacuuugugcgu................................   1       0    seq
    ...................................................................acuuugugcgugugacagu........................   5       0    seq
    ...................................................................acuuugugcgugugacaguga......................   1       0    seq
```

Star   Mature

cauuauaugagacggcgugugcguuggucauugcgcuacgcacaaagaagaugcgcugcuugauuucacuuugugcgugugacagugacaaugcacgcacgucacugcuu			
....acuuugugcgugugacagugG......................	2	1	seq
....acuuugugcgugugacagugac.....................	1	0	seq
....acuuugAgcgugugacagugaca....................	1	1	seq
....acuuugugcgugugacagugaca....................	76	0	seq
....acuuugugcgugugacagugaAa....................	1	1	seq
....Gcuuugugcgugugacagugaca....................	3	1	seq
....acuuugugcgugugacagugacaa...................	44	0	seq
....acuuugugcgugugacagugacaaA..................	1	1	seq
....acuuugugcgugugacagugacaau..................	2	0	seq
....cuuugugcgugugacagugaca....................	1	0	seq
....Auuugugcgugugacagugaca....................	2	1	seq
....cuuugugcgugugacagugacaau..................	3	0	seq
....uuugugcgugugacagug.......................	8	0	seq
....uuugugcgugugacaguga......................	137	0	seq
....uuugugcgugugacagugG......................	20	1	seq
....uuugAgcgugugacaguga......................	1	1	seq
....uuugugcgugugacagugU......................	25	1	seq
....uuugugcgugugacagugaA.....................	6	1	seq
....uuugugcgugugacagugac.....................	157	0	seq
....Auugugcgugugacagugac.....................	1	1	seq
....uuugugcguguCacagugac.....................	1	1	seq
....uuugugcgugugacagugaU.....................	2	1	seq
....uuugugcgAgugacagugac.....................	1	1	seq
....uuugugUgugugacagugac.....................	1	1	seq
....uuugAgcgugugacagugac.....................	1	1	seq
....uuugugcgugugacaAugaca....................	8	1	seq
....uuugugcgugugacagCgaca....................	2	1	seq
....uuugugcgugugacaguCaca....................	4	1	seq
....uuuguCcgugugacagugaca....................	1	1	seq
....uuugugcgugugacagugacN....................	1	1	seq
....uuugugGgugugacagugaca....................	3	1	seq
....uuugugcgugugacagugUca....................	6	1	seq
....uuugugcgugugacaUugaca....................	2	1	seq
....uuugugcgugAgacagugaca....................	4	1	seq
....uuugugcgugugacagugGca....................	5	1	seq
....uuugugAgugugacagugaca....................	48	1	seq
....uuugugcgugugCcagugaca....................	3	1	seq
....uuugugUgugugacagugaca....................	12	1	seq
....uCugugcgugugacagugaca....................	3	1	seq
....uuugugcgugugacagugaUa....................	4	1	seq
....uuugugcguguAacagugaca....................	7	1	seq
....uuugugcgugugacaguUaca....................	2	1	seq
....uAugugcgugugacagugaca....................	22	1	seq
....uuugugcgugugacagugaca....................	28104	0	seq
....uuugugcgAgugacagugaca....................	5	1	seq
....uuugCgcgugugacagugaca....................	1	1	seq
....uuugugcguAugacagugaca....................	3	1	seq
....Auugugcgugugacagugaca....................	69	1	seq
....uuugugcgugugacagugaAa....................	3	1	seq
....uuugugcAugugacagugaca....................	2	1	seq
....uuugugcgugugacagugCca....................	4	1	seq
....uuugugcgugugacagugacU....................	264	1	seq
....uuugugcUugugacagugaca....................	1	1	seq
....uuugugcgugugacaguAaca....................	5	1	seq
....uuugugcgugugacagugacG....................	23	1	seq
....uuugugcgugugacagAgaca....................	13	1	seq
....uuugugcgugugacagugaGa....................	1	1	seq
....uuugugcgugugGcagugaca....................	4	1	seq
....uuuUugcgugugacagugaca....................	2	1	seq
....uuugugcgugugaUagugaca....................	7	1	seq
....Guugugcgugugacagugaca....................	19	1	seq
....uuAgugcgugugacagugaca....................	14	1	seq
....uuugugcgCgugacagugaca....................	3	1	seq
....uuugugcgugugacGgugaca....................	8	1	seq
....uuugugcgugugacUgugaca....................	2	1	seq
....uuugugcCugugacagugaca....................	1	1	seq
....uuugAgcgugugacagugaca....................	22	1	seq
....uuugugcguCugacagugaca....................	2	1	seq
....uuugugcgugugaAagugaca....................	6	1	seq
....uuuguNcgugugacagugaca....................	1	1	seq

```
                          Star                                           Mature
cauuauaugagacggcgugugcguuggucauugcgcuacgcacaaagaagaugcgcugcuugauuucacuuugugcgugugacagugacaaugcacgcacgucacugcuu
.....................................................................Nuugugcgugugacagugaca....................    8     1    seq
.....................................................................uuugugcguguCacagugaca....................    2     1    seq
.....................................................................uuugugcgugugacagNgaca....................    1     1    seq
.....................................................................uuuguAcgugugacagugaca....................    2     1    seq
.....................................................................Cuugugcgugugacagugaca....................    3     1    seq
.....................................................................uuuAugcgugugacagugaca....................    6     1    seq
.....................................................................uuCgugcgugugacagugaca....................    5     1    seq
.....................................................................uuugugcgugCgacagugaca....................    4     1    seq
.....................................................................uuugugcgGgugacagugaca....................    1     1    seq
.....................................................................uuugugcgugugUcagugaca....................    3     1    seq
.....................................................................uAugugcgugugacagugacaa...................    4     1    seq
.....................................................................uuugugcgugugacagAgacaa...................    1     1    seq
.....................................................................uuugugcgugugacagugacaa...................    3162  0    seq
.....................................................................uuugugcgugugaAagugacaa...................    1     1    seq
.....................................................................uuugugcgugugacagugacUa...................    1     1    seq
.....................................................................uuugugcgugugacagugacGa...................    1     1    seq
.....................................................................uuugugcgugugacagugacaG...................    3     1    seq
.....................................................................Auugugcgugugacagugacaa...................    13    1    seq
.....................................................................uuugAgcgugugacagugacaa...................    2     1    seq
.....................................................................uuugugcguguUacagugacaa...................    1     1    seq
.....................................................................uuugugcgugugacagGgacaa...................    1     1    seq
.....................................................................uuugugcguguAacagugacaa...................    4     1    seq
.....................................................................uuugugcgugugacGgugacaa...................    1     1    seq
.....................................................................uuugugcgugugacagugaAaa...................    1     1    seq
.....................................................................uuAgugcgugugacagugacaa...................    2     1    seq
.....................................................................uuugugcAugugacagugacaa...................    1     1    seq
.....................................................................uuugugcgugugacagugacaU...................    22    1    seq
.....................................................................uuugugcgugugacagugGcaa...................    2     1    seq
.....................................................................uuugugcguUugacagugacaa...................    1     1    seq
.....................................................................uuugugcgugAgacagugacaa...................    1     1    seq
.....................................................................uuugugUgugugacagugacaa...................    2     1    seq
.....................................................................Guugugcgugugacagugacaa...................    1     1    seq
.....................................................................uuugugcgugugacaguCacaa...................    1     1    seq
.....................................................................uuuguAcgugugacagugacaa...................    1     1    seq
.....................................................................uuugugAgugugacagugacaa...................    2     1    seq
.....................................................................uuugugAgugugacagugacaau..................    24    1    seq
.....................................................................uuugugcgugugacagCgacaau..................    1     1    seq
.....................................................................uuugugcguguAacagugacaau..................    5     1    seq
.....................................................................uuugugcgugAgacagugacaau..................    4     1    seq
.....................................................................uGugugcgugugacagugacaau..................    1     1    seq
.....................................................................uuugugcgugugacagAgacaau..................    6     1    seq
.....................................................................uuugugcgugugacaguUacaau..................    2     1    seq
.....................................................................uuugugcgugugacagugaGaau..................    3     1    seq
.....................................................................uuugugcgugugacagugacGau..................    7     1    seq
.....................................................................uuugugcgugugacagugaUaau..................    4     1    seq
.....................................................................Guugugcgugugacagugacaau..................    18    1    seq
.....................................................................uuugugcgugugacagugacaUu..................    16    1    seq
.....................................................................uuugugcgAgugacagugacaau..................    9     1    seq
.....................................................................uuugugcgugugacagugUcaau..................    7     1    seq
.....................................................................uCugugcgugugacagugacaau..................    4     1    seq
.....................................................................Auugugcgugugacagugacaau..................    64    1    seq
.....................................................................uuugugcgugugacaguCacaau..................    1     1    seq
.....................................................................uuugugcgugugacagugacUau..................    3     1    seq
.....................................................................uuugugcgugugacagugGcaau..................    7     1    seq
.....................................................................uuugugcguCugacagugacaau..................    3     1    seq
.....................................................................uuugugcAugugacagugacaau..................    4     1    seq
.....................................................................uuAgugcgugugacagugacaau..................    5     1    seq
.....................................................................uuugGgcgugugacagugacaau..................    3     1    seq
.....................................................................uuugugcgugugacagugacaGu..................    3     1    seq
.....................................................................uuugugcgugugacagugacaaC..................    5     1    seq
.....................................................................uuugugcgugugGcagugacaau..................    7     1    seq
.....................................................................uuugugcguAugacagugacaau..................    5     1    seq
.....................................................................Cuugugcgugugacagugacaau..................    11    1    seq
.....................................................................uNugugcgugugacagugacaau..................    1     1    seq
.....................................................................uAugugcgugugacagugacaau..................    14    1    seq
.....................................................................uuugugcgCgugacagugacaau..................    1     1    seq
.....................................................................uuuCugcgugugacagugacaau..................    1     1    seq
.....................................................................uuugugcgugugacGgugacaau..................    2     1    seq
.....................................................................uuugugcguguCacagugacaau..................    3     1    seq
.....................................................................uuuUugcgugugacagugacaau..................    3     1    seq
```

```
                         Star                                         Mature
cauuauaugagacggcgugugcguuggucauugcgcuacgcacaaagaagaugcgcugcuugauuucacuuugugcgugugacagugacaaugcacgcacgucacugcuu
.....................................................................uuugugcgugugacaUugacaau..................   1      1    seq
.....................................................................Nuugugcgugugacagugacaau..................   5      1    seq
.....................................................................uuugugcguguUacagugacaau..................   2      1    seq
.....................................................................uuugugGgugugacagugacaau..................   4      1    seq
.....................................................................uuugugcgugugacagugacaaA..................   331    1    seq
.....................................................................uuugugUgugugacagugacaau..................   10     1    seq
.....................................................................uuugugcgugCgacagugacaau..................   3      1    seq
.....................................................................uuugugcgugugacaguAacaau..................   6      1    seq
.....................................................................uuuAugcgugugacagugacaau..................   9      1    seq
.....................................................................uuugCgcgugugacagugacaau..................   3      1    seq
.....................................................................uuugugcgugugaAagugacaau..................   2      1    seq
.....................................................................uuugugcgugugacagugacCau..................   1      1    seq
.....................................................................uuugugcgugugUcagugacaau..................   5      1    seq
.....................................................................uuugugcgugugacagugaAaau..................   4      1    seq
.....................................................................uuugugcgGgugacagugacaau..................   1      1    seq
.....................................................................uuuguUcgugugacagugacaau..................   1      1    seq
.....................................................................uuuguAcgugugacagugacaau..................   1      1    seq
.....................................................................uuugugcgugugacagugacaau..................   24286  0    seq
.....................................................................uuugugcgugugacUgugacaau..................   3      1    seq
.....................................................................uuugAgcgugugacagugacaau..................   17     1    seq
.....................................................................uuugugcgugugacagugacaauA.................   4810   1    seq
.....................................................................uuugugcgugugacagugacaauU.................   3809   1    seq
.....................................................................uuugugcgugugacagugacaaug.................   4      0    seq
.....................................................................uuugugcgugugacagugacaauC.................   120    1    seq
.....................................................................uuugugcgugugacagugacaaugA................   1      1    seq
.....................................................................uuugugcgugugacagugacaaugc................   2      0    seq
......................................................................uugugcgugugacagugac.....................   5      0    seq
......................................................................uugugcgAgugacagugac.....................   1      1    seq
......................................................................uugugcgugugaAagugaca....................   1      1    seq
......................................................................uugugcgugugacagugacU....................   51     1    seq
......................................................................uugugUgugugacagugaca....................   1      1    seq
......................................................................uugugcAugugacagugaca....................   2      1    seq
......................................................................Cugugcgugugacagugaca....................   1      1    seq
......................................................................uugugcguguUacagugaca....................   1      1    seq
......................................................................uugugcgugugacagugaca....................   1617   0    seq
......................................................................Augugcgugugacagugaca....................   6      1    seq
......................................................................Gugugcgugugacagugaca....................   1      1    seq
......................................................................uugugcgugugacagugaAa....................   1      1    seq
......................................................................uugugcgugugacagugGca....................   2      1    seq
......................................................................uugAgcgugugacagugaca....................   1      1    seq
......................................................................uugugcgugugacagugacG....................   2      1    seq
......................................................................uugugcgugugacaguAacaa...................   1      1    seq
......................................................................uugugcgugugacagugacaa...................   306    0    seq
......................................................................Augugcgugugacagugacaa...................   1      1    seq
......................................................................uugugcgugugacagCgacaa...................   1      1    seq
......................................................................uugugcgugugacagugaUaau..................   2      1    seq
......................................................................uugugcgugugacaguCacaau..................   1      1    seq
......................................................................uugugcgugugacagugGcaau..................   1      1    seq
......................................................................uugugcguUugacagugacaau..................   1      1    seq
......................................................................uugugcgAgugacagugacaau..................   1      1    seq
......................................................................uugugcgugugacagCgacaau..................   2      1    seq
......................................................................uugugcgugugacagugacGau..................   3      1    seq
......................................................................uugugcgCgugacagugacaau..................   4      1    seq
......................................................................uugugcgugugaGagugacaau..................   1      1    seq
......................................................................uugugcgugugacaguAacaau..................   4      1    seq
......................................................................uugugAgugugacagugacaau..................   9      1    seq
......................................................................uugugcgugugGcagugacaau..................   2      1    seq
......................................................................uugugUgugugacagugacaau..................   5      1    seq
......................................................................uuAugcgugugacagugacaau..................   1      1    seq
......................................................................uuCugcgugugacagugacaau..................   1      1    seq
......................................................................uugugcgugugacagugacaUu..................   28     1    seq
......................................................................Gugugcgugugacagugacaau..................   2      1    seq
......................................................................uugugcgugugacagugacaaA..................   11     1    seq
......................................................................Cugugcgugugacagugacaau..................   1      1    seq
......................................................................uAgugcgugugacagugacaau..................   3      1    seq
......................................................................uugugcgugugacagugaGaau..................   1      1    seq
......................................................................uugugcgugugacagugacaaG..................   2      1    seq
......................................................................Nugugcgugugacagugacaau..................   3      1    seq
......................................................................uugugcgugAgacagugacaau..................   5      1    seq
......................................................................uugugcgugugacagugaAaau..................   1      1    seq
```

Star Mature

```
cauuauaugagacggcgugugcguuggucauugcgcuacgcacaaagaagaugcgcugcuugauuucacuuugugcgugugacagugacaaugcacgcacgucacugcuu
......................................................................uuguAcgugugacagugacaau..................   1      1   seq
......................................................................uugugcgugugacGgugacaau..................   3      1   seq
......................................................................uugugcgugCgacagugacaau..................   1      1   seq
......................................................................uugCgcgugugacagugacaau..................   2      1   seq
......................................................................uugugcgugugacagAgacaau..................   6      1   seq
......................................................................uugugcgugugacagugacaau..................   5803   0   seq
......................................................................uugugcgugugacagugacaaC..................   2      1   seq
......................................................................uugugcUugugacagugacaau..................   2      1   seq
......................................................................uugAgcgugugacagugacaau..................   2      1   seq
......................................................................uugugcguCugacagugacaau..................   1      1   seq
......................................................................uugGgcgugugacagugacaau..................   2      1   seq
......................................................................uugugcgugugUcagugacaau..................   2      1   seq
......................................................................uugugcgugugacagugUcaau..................   3      1   seq
......................................................................Augugcgugugacagugacaau..................   11     1   seq
......................................................................uugugcgugugacagugacaauC.................   2      1   seq
......................................................................uugugcgugugacagugacaauU.................   266    1   seq
......................................................................uugugcgugugacagugacaauA.................   157    1   seq
......................................................................uugugcgugugacagugacaauAc................   1      1   seq
.......................................................................ugugcgugugacagugaca....................   11     0   seq
.......................................................................ugugcgugugacagAgacaau..................   2      1   seq
.......................................................................ugugcgugugacagugacaau..................   116    0   seq
.......................................................................ugugcgugugacagugacaaug.................   27     0   seq
.......................................................................ugugcgugugacagugacaauC.................   3      1   seq
.......................................................................ugugcgugugacagugacaauU.................   672    1   seq
.......................................................................ugugcgugugacagugacaauA.................   40     1   seq
.......................................................................ugugcgugugacagugacaaugc................   1      0   seq
.........................................................................ugcgugugacagugacaaugca...............   3      0   seq
```
